# Sensitive and accurate analysis of gene expression signatures enabled by oligonucleotide-labelled cDNA

**DOI:** 10.1080/15476286.2022.2078093

**Published:** 2022-06-02

**Authors:** Žana Kapustina, Justina Medžiūnė, Varvara Dubovskaja, Karolis Matjošaitis, Simona Žeimytė, Arvydas Lubys

**Affiliations:** a Thermo Fisher Scientific Baltics, Research and Development Department, Vilnius, Lithuania; b Faculty of Chemistry and Geosciences, Vilnius University, Vilnius, Lithuania

**Keywords:** RNA-seq, transcriptomics, modified nucleotides, library preparation, 3’-end sequencing, gene expression profiling

## Abstract

High-throughput RNA sequencing offers a comprehensive analysis of transcriptome complexity originated from regulatory events, such as differential gene expression, alternative polyadenylation and others, and allows the increase in diagnostic capacity and precision. For gene expression profiling applications that do not specifically require information on alternative splicing events, the mRNA 3′ termini counting approach is a cost-effective alternative to whole transcriptome sequencing. Here, we report MTAS-seq (mRNA sequencing via terminator-assisted synthesis) – a novel RNA-seq library preparation method directed towards mRNA 3′ termini. We demonstrate the specific enrichment for 3′-terminal regions by simple and quick single-tube protocol with built-in molecular barcoding to enable accurate estimation of transcript abundance. To achieve that, we synthesized oligonucleotide-modified dideoxynucleotides which enable the generation of cDNA libraries at the reverse transcription step. We validated the performance of MTAS-seq on well-characterized reference bulk RNA and further tested it with eukaryotic cell lysates.

## Introduction

Most isogenic cells of multicellular organisms produce transcripts with a complex spatial, temporal and structural variety. RNA abundance regulation and alternative processing play a central role in shaping phenotypic complexity, with more than 90% of human genes undergoing alternative splicing and nearly 70% having multiple polyadenylation sites [1–3].

Advances in sequencing technologies empower to study quantitative and structural aspects of RNA biology down to a single-nucleotide level resolution from inputs as low as the contents of individual cells [4,5]. Whole transcriptome sequencing generates the most comprehensive transcriptomic datasets; however, the sensitivity and accuracy of detection of relative changes in gene expression across sample groups are hindered by read coverage bias towards longer transcripts [6,7]. While long-read sequencing technologies which allow full-length transcript analysis, such as Iso-seq, may solve this issue by producing a single read per transcript with no tradeoff in regards to structural information, currently this approach is mostly adopted to study non-model organisms [8,9]. For digital gene expression profiling on short-read sequencers, library preparation techniques that generate only one fragment per transcript either at the 5′ or 3′ terminus are acknowledged as a good cost-effective alternative to whole transcriptome RNA-seq and were rapidly adopted for high-throughput single-cell sequencing [10–12].

Different types of noise which are usually classified as either technical or biological by origin influence quantitative results in RNA-seq [13]. Technical noise includes variation caused by the laboratory manipulations, from RNA extraction to sequencing. It was reported that different RNA extraction procedures substantially affect relative transcript abundance [14,15]. While in single-cell studies RNA extraction is avoided for obvious reasons, robust bulk library preparation directly from crude lysates could reduce ‘batch effects’ and improve the quality of meta-analyses. In addition, PCR amplifies different molecules with unequal efficiencies. Labelling each cDNA fragment with a unique molecular barcode provides an absolute scale of measurement that helps to remove PCR-induced artefacts accurately, while removing duplicates without molecular barcodes might eliminate many biologically meaningful reads [16,17].

We have previously shown that introduction of sequencing adapters via enzymatic incorporation of base-modified dideoxynucleotides into nascent DNA can substantially improve the informativeness of 16S rRNA sequencing for the characterization of microbial communities [18]. Here, we describe a new method for high-throughput gene expression profiling that generates fragment libraries from the 3′-terminal transcript regions with the rapid and simple single-tube protocol. We termed this approach mRNA sequencing via terminator-assisted synthesis or MTAS-seq. The novel technique for cDNA labelling with artificial sequences developed in this work allowed us to integrate fragmentation and adapter addition into a single enzymatic step. We validated MTAS-seq by sequencing well-characterized RNA and synthetic standards. Moreover, we showed the compatibility of our method with library preparation from crude cell lysates.

## Results

### Modified dideoxynucleotides enable the generation of high-quality libraries of oligonucleotide-labelled cDNAs

**Figure 1.**
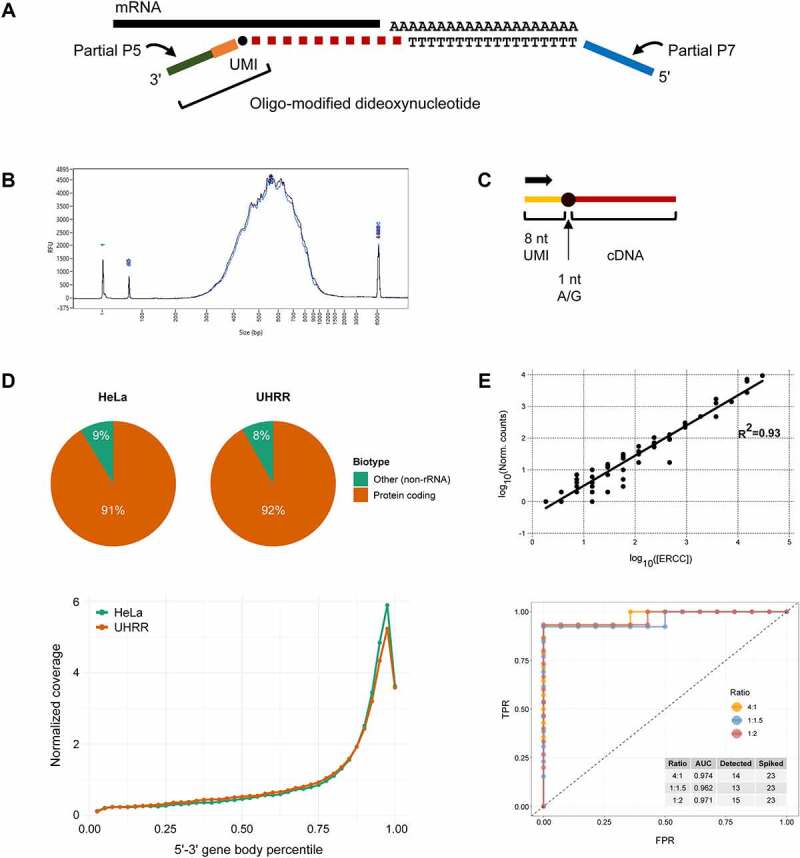
Overview of MTAS-seq technique. (A) Reverse transcription starts from an oligo (dT) primer containing a portion of the Illumina P7 adapter sequence. Primer extension terminates upon the incorporation of oligonucleotide-modified dideoxynucleotide bearing a portion of the Illumina P5 adapter sequence. This yields cDNA fragments which can be PCR-amplified using standard Illumina indexing primers. (B) A typical MTAS-seq library trace. (C) The structure of sequencing reads is as follows: 8 nt UMI sequence followed by a nucleotide complementary to the incorporated terminator (two or more bases are expected to appear at the indicated position if a mixture of OTDDNs with different nucleobases is used at the reverse transcription step) and a portion of 3′ UTR. (D) RNA species captured in MTAS-seq libraries prepared from well-characterized RNA and typical gene body coverage. Note that apart from non-mRNA transcript species, such as lincRNAs, ‘Other’ category includes ERCC RNA Spike-Ins which were captured via their polyA tails. (E) The correlation coefficient (R^2^) of detected ERCC counts versus expected in MTAS-seq library prepared from 500 ng of UHRR with ~2% of ERCC mix was 0.93, with 55 different ERCCs detected. ROC curves indicate *erccdashboard* analysis to assess the performance of differential expression estimation. TPR – true positive rate, FPR – false positive rate.

We developed MTAS-seq (Figure 1A), which leverages a nucleotide-mediated adapter addition technology for rapid and simple transcriptome-wide differential expression profiling and 3′UTR detection. Reverse transcription primer targets polyA tails of eukaryotic mRNAs and is extended by reverse transcriptase. Primer extension is terminated by stochastic incorporation of oligonucleotide-tethered dideoxynucleotides (OTDDNs) yielding oligonucleotide-labelled cDNA fragments (Figure 1B) whose average length is determined by the ratio of OTDDNs to respective dNTPs. This step executes two library prep prerequisites – fragmentation and adapter addition – at once. Importantly, the resulting cDNA fragments, having an unnatural linkage within OTDDN, are biocompatible, i.e. they are suitable for standard PCR which amplifies these fragments and introduces full-length NGS platform-specific adapters. We optimized the workflow such as to eliminate the need for intermediate purification of cDNA fragments before amplification (see Methods), thus making the library preparation a single-tube process with no material losses throughout the procedure. Moreover, OTDDNs used in this work contain oligonucleotide modification with a region of randomized sequence which serves as a unique molecular identifier (UMI) to enable accurate elimination of PCR noise from sequencing data. As a result, the sequencing read starts with an in-line UMI, followed by a cDNA fragment (Figure 1C).

The use of ‘click’ chemistry as a mechanism of adapter addition that follows terminated polymerization reaction was previously reported [19,20]. Here, we executed a ‘click’ reaction to generate OTDDNs with oligonucleotides attached to the nucleobases of dideoxynucleotides via their 5′ termini before their incorporation into cDNA. We obtained base-modified conjugates with ≥97% purity and ≥20% yield. We optimized the structure of an unnatural linker and identified reverse transcriptases able to use OTDDNs as substrates as well as polymerases able to perform read-through [21]. This technology enables straightforward cDNA labelling with any desired synthetic oligonucleotide and easy addition of unique molecular labels.

To validate the technique, we sequenced MTAS-seq libraries prepared from well-characterized HeLa and UHRR total RNA samples spiked with ERCC transcript mixes, with three technical replicates per RNA input, which ranged from 0.5 ng to 500 ng. The obtained libraries were of similar size indicating that OTDDN incorporation rate is robust across different RNA inputs given the same OTDDN ratio to corresponding dNTPs (Fig. S1). 99.4–99.8% of sequencing reads from each sample mapped to the human genome and ERCCs after UMI trimming, with strand specificity of >99%. Importantly, on average 94.5% of reads were uniquely mapped in HeLa samples, and 93.4% were in UHRR samples. The high percentage of uniquely aligned reads might be attributed to rather long insert sizes of MTAS-seq libraries. We obtained sequences for more than 19,000 genes in UHRR samples and nearly 15,000 genes in HeLa samples with only 2 M reads. Reverse transcription conditions demonstrated high specificity for mRNAs even though starting material was total RNA: there were virtually no traces of rRNA reads indicating no mispriming events and read coverage, as expected, concentrated at the 3′ terminal region of RNA transcripts (Figure 1D).

To assess the quantitative accuracy of MTAS-seq, we compared the detected ERCC counts to expected ones and observed that with at least 50 unique ERCC transcripts identified, the correlation (R^2^) with the expected distribution is 0.91–0.94. We next evaluated the discriminatory power of differential expression detection by assessing ERCC ratio detection performance with receiver operating characteristic (ROC) curves and area under the curve (AUC) statistics. With at least 13 ERCC spikes detected per abundance ratio, AUC analysis indicated good diagnostic power of MTAS-seq assay, with AUC values >0.96 for all ratios (Figure 1E). This suggests the utility of MTAS-seq for highly accurate gene expression profiling, with an additional advantage of UMI labelling which is especially important for low-input applications prone to high PCR duplication rates (Fig. S2).

### MTAS-seq allows transcriptional profiling directly from cell lysates

To assess whether high-quality libraries might be produced directly from eukaryotic cell lysates leaving behind RNA extraction, we first purified total RNA from a known number of HEK-293 cells and determined the approximate amount of RNA per cell, which was ~12 pg. Next, we prepared MTAS-seq libraries from various amounts of HEK-293 cells (see Methods) and, in parallel, from purified bulk RNA which amount corresponded to the cellular RNA contents used in the crude lysate experiment. Libraries for each input were prepared in quadruplicates.

**Figure 2.**
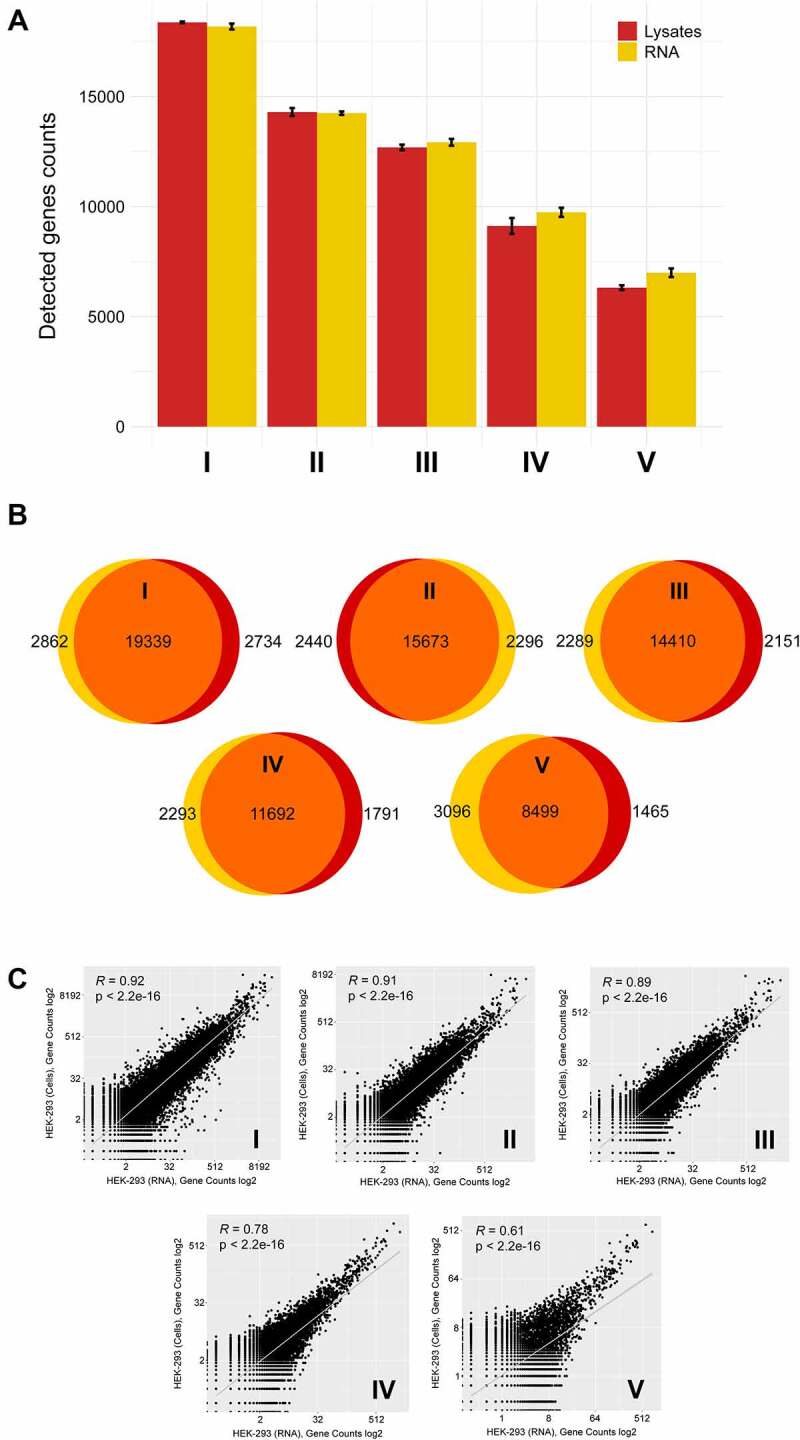
Gene expression profiling in HEK-293 total RNA and crude cell lysates. (A) Average numbers of detected genes in MTAS-seq libraries prepared from different amounts of RNA and cells. Error bars represent the standard error of the mean (SEM). (B) Venn diagrams depict the overlap of detected genes at a uniform read depth. Red circles correspond to crude lysate samples, while yellow circles correspond to bulk RNA samples. (C) Correlation of gene counts of corresponding RNA and lysate samples. I – 120 ng RNA or 10,000 cells, II – 12 ng RNA or 1,000 cells, III – 6 ng RNA or 500 cells, IV – 1.2 ng RNA or 100 cells, V – 0.12 ng RNA or 10 cells.

On average, 99.1% (98.6–99.3%) of sequencing reads aligned to the human genome in crude lysate samples, and 99.2% (96.7–99.5%) aligned to the human genome in purified RNA samples. The percentages of uniquely mapped reads on average were 92.8% for crude lysates and 94.5% for purified RNA. We observed a good agreement of gene detection capacity between corresponding lysate and RNA samples (Figure 2 A and B) as well as strong technical reproducibility of data obtained from crude lysates (Fig. S3). Moreover, gene counts strongly correlated between corresponding lysate and RNA samples, with Spearman′s correlation coefficient values of >0.7 for input amounts as small as 1.2 ng of total RNA or 100 cells (Figure 2C). Notably, data variability obtained with 0.12 ng of total RNA or 10 cells was greater than that for higher input amounts. This is an expected effect that was previously observed in RNA sequencing studies [22]. We further applied the direct library preparation approach for a different cell type – mouse BALB/3T3 fibroblasts – and obtained high-quality data confirming the reliability and robustness of MTAS-seq as well as the ability to generate libraries from sub-nanogram quantities of total RNA (Fig. S4). Such flexibility allows to analyse even limiting samples for which RNA extraction is impractical.

## Discussion

Despite the decreasing cost of next-generation sequencing, sample preparation remains expensive and can be prohibitive for large-scale experimental studies; thus, newly proposed library generation methods are expected to be both reliable and cost-effective. Here, we have demonstrated that the use of OTDDNs enables the simple and quick generation of cDNA libraries enriched for mRNA 3′-terminal sequences and reduces the number of workflow steps. Moreover, randomized sequence within the oligonucleotide modification tags each fragment with a unique barcode which in turn contributes to highly accurate estimation of transcript abundance in sequencing data.

MTAS-seq approach can be viewed as a simplified version of chemoenzymatic library preparation, such as Poly(A)-ClickSeq [23]. Although Poly(A)-ClickSeq procedure is relatively easy to execute, separate chemical ligation step does not allow the development of a single-tube protocol and requires an intermediate purification step which inevitably leads to the loss of material. The authors demonstrated that 125 ng of total RNA is minimally required to generate a library, while single-tube MTAS-seq was able to process sub-nanogram quantities of starting material. Finally, Poly(A)-ClickSeq generated ~50% of usable reads, while >99% of MTAS-seq reads were aligned to the reference genome and processed further. This illustrates the superior technical characteristics of MTAS-seq, retaining all general benefits of 3′ mRNA sequencing approach.

Gene expression analysis strives for minimal perturbation of the original cellular RNA content during sample preparation. Previous studies revealed the feasibility of reverse transcription directly on cell lysates [24], and these findings have been applied to scRNA-seq [25]. Notably, direct reverse transcription on cell lysates has not been implemented in bulk RNA-seq until recently. An attempt to combine direct cDNA synthesis with Smart-3SEQ was reported to generate high-quality data as compared to library preparation from extracted RNA [26]. Similarly, MTAS-seq demonstrated an equivalent performance with cell lysates and purified total RNA suggesting the possibility to eliminate the RNA purification step without compromising data quality.

Overall, MTAS-seq provides an accurate and efficient method for high-throughput gene expression profiling with the following highlights: (i) simple single-tube library preparation protocol exhibits solid performance in the range of 0.12–500 ng of total RNA; (ii) easy UMI labelling ensures bias correction for highly accurate estimation of transcript abundance and differential expression; (iii) protocol does not require RNA pre-processing, i.e. enrichment/depletion and fragmentation steps, before library generation and (iv) workflow is compatible with reverse transcription on cell lysates. This approach might greatly facilitate gene expression profiling studies to unravel molecular signatures of complex diseases. Further development of MTAS-seq could include the investigation of its broader applicability both in terms of compatible sample types, e.g. including degraded RNA samples and single-cell analysis protocols, and in terms of analytical capabilities, such as providing information on alternative polyadenylation.

## Methods

### Synthesis of oligonucleotide-tethered dideoxynucleotides

All reaction components were added to the reaction mixture as solutions in water unless specified otherwise. Modified oligonucleotide 5′-hexynyl-NNNNNNNNAGATCGGAAGAGCGTCGTGTAGGGAAAGAG-phosphate-3′ (ON) was used for coupling to dideoxynucleotides. All oligonucleotides used in this work were synthesized by Metabion GmbH requesting HPLC purification.

5-(3-(2-Azidoacetamido)prop-1-ynyl)-2’,3’-dideoxycytidine-5′-triphosphate or 5-(3-(2-azidoacetamido)prop-1-ynyl)-2’,3’-dideoxyuridine-5′-triphosphate (3 eq.) solution was added to 5′-hexynyl-modified oligonucleotide (200–210 nmol) solution in sodium phosphate buffer (1 ml, 100 mM, pH 7). A premixed solution of CuSO_4_ (100 mM, 12 eq.) and THPTA (250 mM, 5 eq. to CuSO_4_) was then added to the reaction mixture, followed by the addition of sodium ascorbate (1 M, 50 eq. to CuSO_4_). The reaction mixture was stirred for 20 min at 42°C, quenched with 0.5 M EDTA-Na_2_ solution (1 ml, pH 8). The products were purified by C18 reversed-phase chromatography using 100 mM TEAAc/ACN (11–18%) as eluent and desalted using water/ACN (0–100%) as eluent.

The oligo-modified ddC^ON^TP was obtained with 37% (78 nmol) yield. HRMS (ESI^−^): calculated 39 nt 8 N random oligonucleotide mean mass [M]: 12,743.080; found: 12,743.088. The oligo-modified ddU^ON^TP product was obtained with 20% (40 nmol) yield. HRMS (ESI^−^): calculated 39 nt 8 N random oligonucleotide mean mass [M]: 12,744.064; found: 12,744.063.

The general scheme of OTDDN synthesis is depicted in Fig. S5.

### MTAS-seq library preparation from total RNA and cell lysates

*Samples*. Universal Human Reference RNA and HeLa total RNA were used for proof-of-principle experiments. Invitrogen™ ERCC ExFold RNA Spike-In Mixes (Thermo Fisher Scientific) were used as external controls. Library preparation from cell lysates was performed using HEK-293 (ATCC CRL-1573) and BALB/3T3 (ATCC CCL-163) cells. To compare the library preparation performance using cell lysates and purified RNA, total RNA was extracted from 1 million HEK-293 cells using the Invitrogen™ PureLink™ RNA Mini Kit (Thermo Fisher Scientific) according to the manufacturer′s instructions. RNA quality was assessed by Agilent 2100 Bioanalyzer™ using the RNA 6000 Pico Kit (Agilent Technologies). RNA concentration was measured by NanoDrop™ 2000 Spectrophotometer (Thermo Fisher Scientific).

*Cell cultivation*. Cells were cultured according to the standard mammalian tissue culture protocols and sterile technique. HEK-293 cell line was maintained in Dulbecco′s Modified Eagle's Medium (DMEM) supplemented with 2 mM L-glutamine, 10% foetal bovine serum, 1% gentamicin and 0.00028% β-mercaptoethanol (Thermo Fisher Scientific). BALB/3T3 cells were cultured in DMEM supplemented with 2 mM L-glutamine, 10% donor-sourced bovine serum and 1% gentamicin (Thermo Fisher Scientific). Cells were incubated in a humidified atmosphere of 5% CO_2_ and 95% air at 37°C.

*MTAS-seq library preparation*. Library preparation does not require rRNA depletion or mRNA enrichment as the RT primer effectively selects for polyadenylated transcripts. 0.1–500 ng of total RNA or 10–10,000 cells were used to generate libraries. Reverse transcription was performed in 20 µl reaction mixture containing 200 U of SuperScript™ IV reverse transcriptase (Thermo Fisher Scientific), 50 pmol of RT primer of sequence 5′-CTGGAGTTCAGACGTGTGCTCTTCCGATCT(T)_30_–3′, 20 pmol of dNTP mix, 40 U of RiboLock™ RNase Inhibitor (Thermo Fisher Scientific), 5 mM DTT, 2 pmol of ddU^ON^TP, 0.4 pmol of ddC^ON^TP in 1× of SuperScript IV RT buffer (Thermo Fisher Scientific). For library preparation from cell lysates, reverse transcription reaction was supplemented with 0.3% IGEPAL™ CA-630 (Sigma-Aldrich) to ensure cell lysis. The reaction was performed for 30 min at 50°C followed by termination at 80°C for 10 min. After reverse transcription, the reaction mixture was used directly for cDNA amplification. Reverse transcription reaction was supplemented with 25 µl of Invitrogen™ Collibri™ Library Amplification Master Mix (Thermo Fisher Scientific), 20 U of 3′-5′ exonuclease-deficient Phusion polymerase (Thermo Fisher Scientific) and 50 pmol of each of the unique dual indexing primers:

i5 primer: 5′-AATGATACGGCGACCACCGAGATCTACAC[index]ACACTCTTTCCCTACACGACGCTCTTCCGATCT-3′

i7 primer: 5′-CAAGCAGAAGACGGCATACGAGAT[index]GTGACTGGAGTTCAGACGTGTGCTCTTCCGATCT-3′

Cycling was performed as follows: denaturation at 98°C for 30 s, followed by 10–25 cycles of denaturation at 98°C for 10 s, annealing at 60°C for 30 s, extension at 72°C for 1 min and final extension at 72°C for 1 min. Each PCR reaction was then purified using Dynabeads™ Cleanup Beads (Thermo Fisher Scientific). DNA binding to the beads was performed by mixing 45 µl of bead suspension with 50 µl of sample and subsequent incubation at room temperature for 5 min. The sample was then placed on a magnet, the supernatant was removed and beads were resuspended in 50 µl of elution buffer containing 10 mM Tris-HCl (pH 8.0). 50 µl of fresh beads was added again to the sample and binding was repeated. After room temperature incubation, the sample was placed on a magnet, the supernatant was removed and beads were washed twice with 85% ethanol. To elute libraries, beads were resuspended in 15 µl of elution buffer and incubated for 1 min at room temperature.

To generate enough material for sequencing, low RNA inputs (0.1–0.5 ng) required an additional amplification step. Reamplification was performed in a 50 µl reaction with Invitrogen™ Collibri™ Library Amplification Master Mix with Primer Mix (Thermo Fisher Scientific) for 6–12 cycles according to the recommended temperature conditions. Final libraries were purified using Dynabeads™ Cleanup Beads (Thermo Fisher Scientific) as described above. Fragment size distribution was then assessed by Agilent Fragment Analyzer™ system with HS NGS Fragment kit or Agilent 2100 Bioanalyzer™ with High Sensitivity DNA kit (Agilent Technologies). Quantification of sequenceable molecules was performed with Invitrogen™ Collibri™ Library Quantification Kit (Thermo Fisher Scientific).

### Sequencing

For library quality control, 1 × 150 bp SE sequencing was performed on the Illumina MiSeq™ instrument using MiSeq Reagent Kit v3 (150-cycle). Deep sequencing was performed on the Illumina NovaSeq™ 6000 System (2 × 150 bp PE, pooled libraries were mixed with 20% of PhiX control), aiming for ≥2 M reads per sample.

### Data analysis

All NGS data analysis workflows were implemented using the Snakemake workflow manager v6.1.0 [27].

Quality control for the PE raw reads obtained upon sequencing with the NovaSeq system was performed with BBDuk tool from BBMap suit v37.90 [28] to trim adaptor sequences and exclude low-quality reads and poor-quality bases with the following settings: minlength 50, minquality 30, qtrim r, trimq 15, tpe tbo, maxns 1, ftl = 1, hdist 1, ktrim r, k 23, mink 11. Unique Molecular Identifiers (UMIs) were extracted and analysed with UMI-tools v1.0.1 [29]. After trimming, processed reads were subsampled to 2 M reads with SeqKit v0.1.0 [30] using command line ‘seqkit sample -s 11 – two-pass -n 2,000,000’. The same number of reads for all samples was used as an input for the alignment step. Processed reads were aligned to a reference human genome version hg38 using the Spliced Transcripts Alignment to a Reference (STAR v2.5.3) software [31] with default settings, and then, mapping quality was assessed with Picard v2.22.3 [32], RSeQC v2.6.2 [33] and Qualimap v2.2.1 [34]. For transcript quantification, QORTS v1.3.0 for detected gene counts and pairwise correlations and FeatureCounts v1.6.4 [35] for ERCCdashboard analysis were used. Normalization and differential gene expression analysis was performed in DESeq2 v1.32.0 [36]. External RNA Controls Consortium (ERCC) spike-in ratio mixtures were analysed with the erccdashboard v1.20.0 [37] to collect technical performance metrics.

## Supplementary Material

Supplemental Material

## Data Availability

Sequencing data have been deposited in NCBI BioProject database under the accession number PRJNA768357.

## Code availability

The code used for data analysis is available upon request from the authors.
