# Supplementary material for: Sensitive and accurate analysis of gene expression signatures enabled by oligonucleotide-labelled cDNA
Source: RNA Biol. 2022 Jun 2;19(1):774–80. doi: 10.1080/15476286.2022.2078093 (PMC9191874; doi:10.1080/15476286.2022.2078093)
Supplement: Supplemental Material [file KRNB_A_2078093_SM4368.zip › mRNAseq_Supplementary_Material_RB_REV_clean.docx]

**Supplementary Information**

Sensitive and accurate analysis of gene expression signatures enabled by oligonucleotide-labeled cDNA

Žana Kapustina^1,3*^, Justina Medžiūnė^1,2,3^, Varvara Dubovskaja^1^, Karolis Matjošaitis^1^, Simona Žeimytė^1^ and Arvydas Lubys^1^**^*^**

^1^Thermo Fisher Scientific Baltics, V. A. Graičiūno 8, LT-02241 Vilnius, Lithuania

^2^ Faculty of Chemistry and Geosciences, Vilnius University, Naugarduko str. 24, LT-03225 Vilnius, Lithuania

^3^These authors contributed equally to this work.

**^*^**Correspondence to Ž.K. or A.L. E-mail: zana@olimpiados.lt; arvydas.lubys@thermofisher.com

**
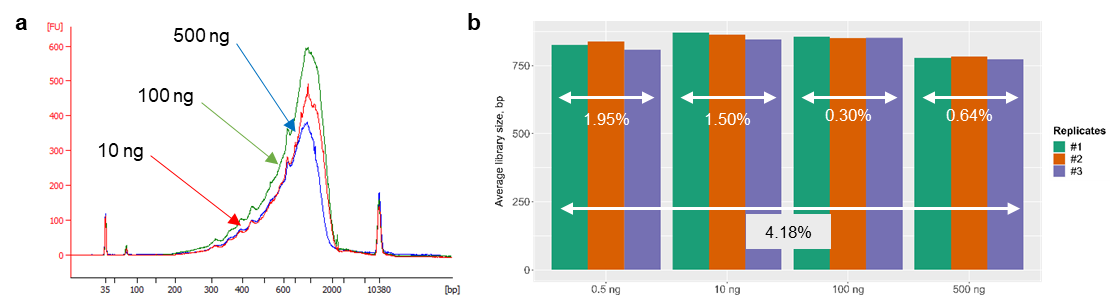
**

**Figure S1. The robustness of oligo-modified terminator incorporation rate across various RNA inputs. a,** MTAS-seq generates libraries of very similar traces from various amounts of total RNA. **b,** The coefficients of variation of the average library size across different RNA inputs and technical replicates.

The data presented here was generated using the following ratios of OTDDNs to respective dNTPs: ddU^ON^TP to dTTP ratio of 1:10 and ddC^ON^TP to dCTP ratio of 1:50, respectively. For more experimental details refer to the Methods section.

**
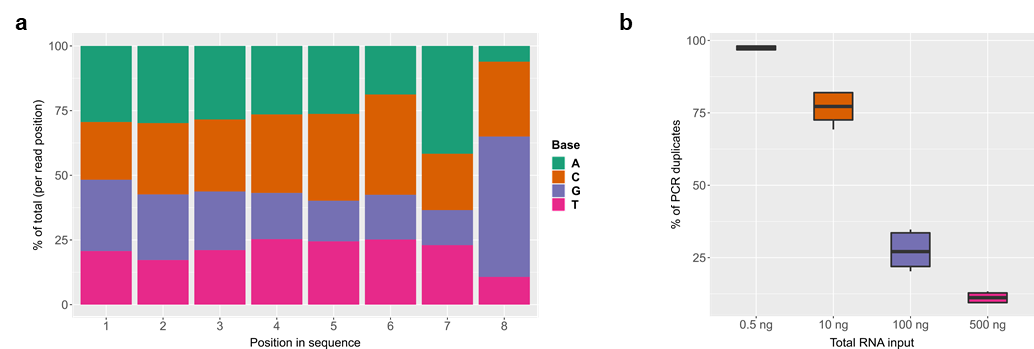
**

**Figure S2. OTDDNs may serve to tag individual cDNA molecules with UMI. a,** Typical base composition within UMI region. **b,** Fraction of PCR duplicates identified by UMIs across genes for a series of MTAS-seq libraries prepared from different amounts of starting material.


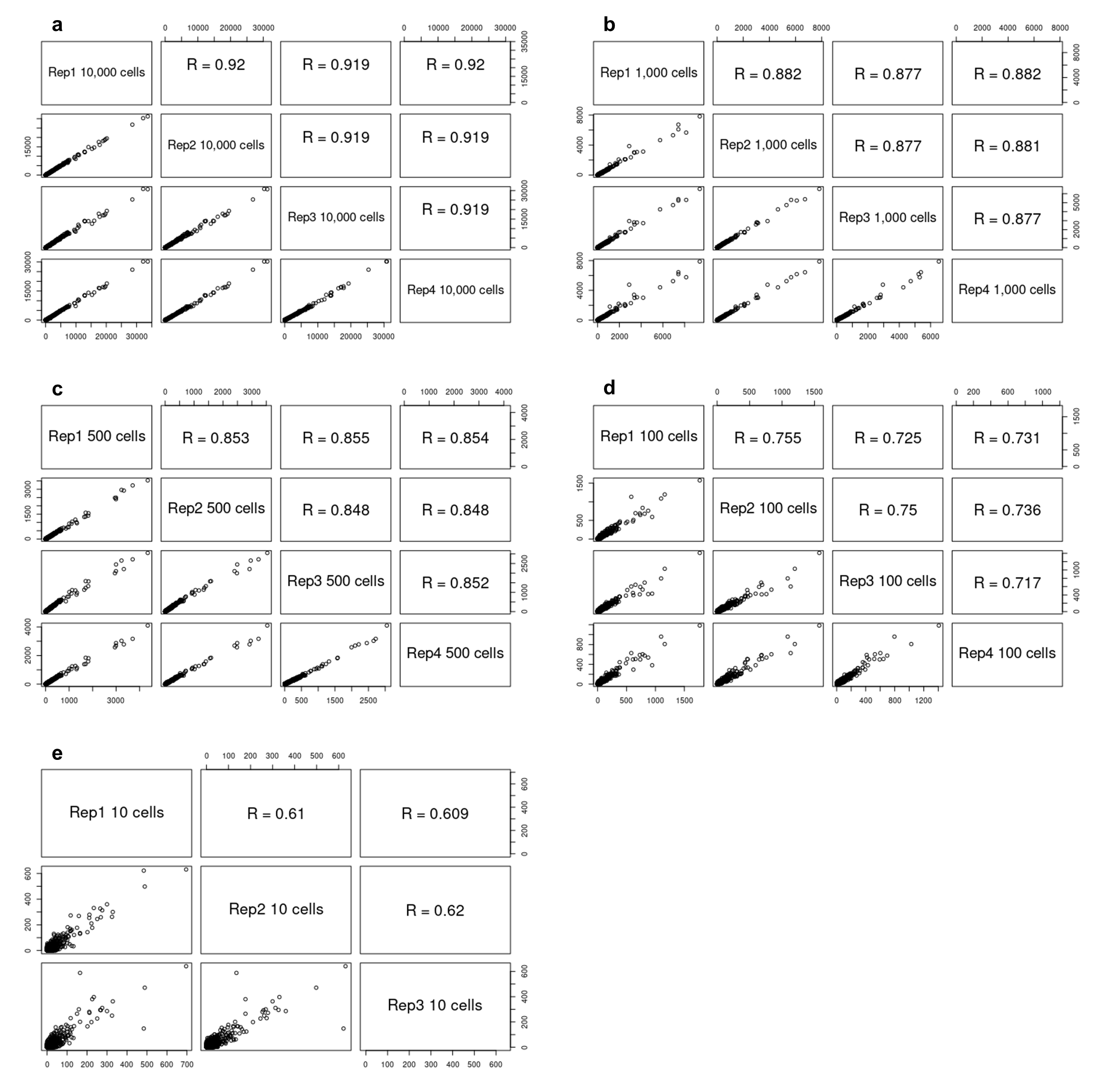


**Figure S3. Gene counts correlation matrices between technical replicates of MTAS-seq libraries prepared from HEK-293 cell lysates. a,** Correlation matrix between 4 technical replicates of MTAS-seq libraries prepared from 10,000 cells. **b,** Correlation matrix between 4 technical replicates of MTAS-seq libraries prepared from 1,000 cells. **c,** Correlation matrix between 4 technical replicates of MTAS-seq libraries prepared from 500 cells. **d,** Correlation matrix between 4 technical replicates of MTAS-seq libraries prepared from 100 cells. **e,** Correlation matrix between 3 technical replicates of MTAS-seq libraries prepared from 10 cells. In all cases, Rep1, Rep2, Rep3 and Rep4 indicate individual replicates. R values correspond to Spearman′s correlation coefficient.

**
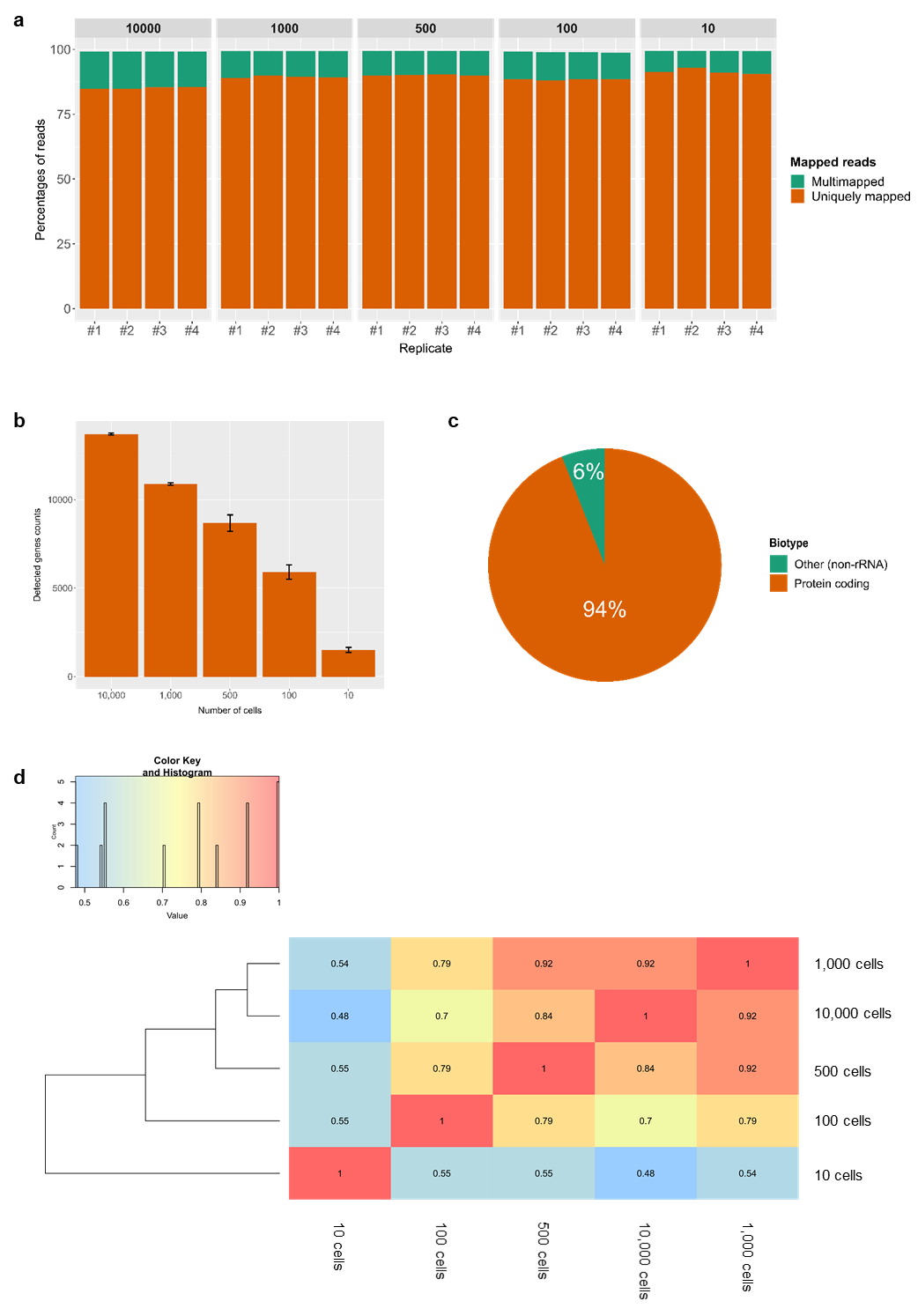
**

**Figure S4. Gene expression profiling in BALB/3T3 cell lysates by MTAS-seq. a,** The percentages of reads aligned to mouse genome in libraries prepared from various amounts of cells. The average amount of uniquely aligned reads was 90.1%. **b,** Average numbers of detected genes in libraries prepared from different amounts of starting material. Error bars represent standard error of the mean (SEM). **c,** Captured RNA species. “Other” category includes lincRNA, pseudogenes and other non-mRNA transcript species. **d,** Gene counts correlation matrix. Mean counts of 4 technical replicates were used for each sample type. Numbers indicate Spearman′s correlation coefficients.


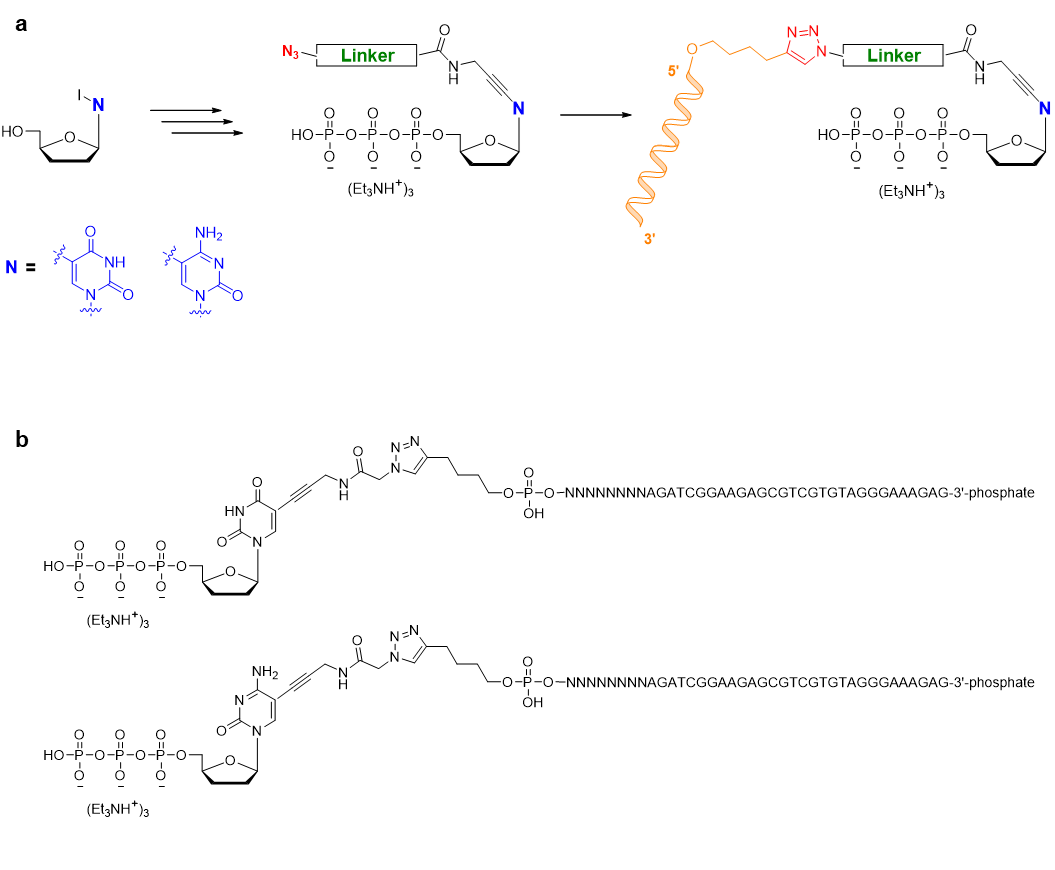


**Figure S5. The scheme of oligonucleotide-tethered dideoxynucleotide (OTDDN) synthesis. a,** The hexynyl-modified oligonucleotide is conjugated to azide-modified dideoxynucleotides using “click” chemistry. **b,** The structures of UMI-containing OTDDNs used in this work for bulk RNA-seq library preparation.
